# COVID-19 Pandemic Impact on Cardiometabolic Markers in Adults in Chongqing, China: A Retrospective Cohort Study

**DOI:** 10.3389/fpubh.2022.859488

**Published:** 2022-06-03

**Authors:** Zhongxiang He, Yong Zhang, XiaoYang Xu, Ying Mei, Jamal Rahmani, Shaikh Sanjid Seraj, XiaoYa Qi

**Affiliations:** ^1^Department of Health Management, The Second Hospital Affiliated With Chongqing Medical University, Chongqing, China; ^2^School of Public Health and Health Management, Chongqing Medical University, Chongqing, China; ^3^Cancer Research Center, Shahid Beheshti University of Medical Sciences, Tehran, Iran; ^4^Walsall Manor Hospital, Walsall Healthcare NHS Trust, Walsall, United Kingdom

**Keywords:** COVID-19, cardiometabolic profiles, obesity, sedentary lifestyle, lockdown

## Abstract

The influx of COVID-19 infection and government-enforced lockdowns and social isolation changed people's lifestyles. Concerns regarding the health impact of the COVID-19 pandemic due to the new sedentary lifestyle. This study aims to investigate the impact of the COVID-19 pandemic on cardiovascular health factors. A retrospective observational study was conducted using historical medical records. The cohort consisted of healthy adults (without chronic non-communicable diseases) over 18 years of age who have undertaken a health examination at the Chongqing Medical University from 2019 to 2020. The analysis of covariance (ANCOVA) test was used to compare variables between 2019 and 2020. The effect of exposure time to COVID-19 on cardiometabolic markers was analyzed using multiple linear regression models. 29,773 participants took part in this study. The average age was 42.5 ± 13.44 years at baseline, and the average follow-up period was 12.7 ± 2.8 months. Analysis showed that weight, BMI, waist circumference, hip circumference, WHR, fasting blood glucose, TG, LDL, uric acid, and liver enzymes increased significantly during the COVID-19 pandemic (*P* < 0.05). This study showed evidence that the COVID-19 pandemic and its control measures negatively impacted cardiometabolic profiles.

## Introduction

More than 200 million confirmed cases and more than 5 million deaths have been estimated to be caused by COVID-19 at the end of 2021 (1).

China has taken strict measures to prevent COVID-19 from spreading in the community, including city lockdowns, quarantines, social-distance orders, stay-at-home orders, and travel restrictions (2, 3). Government-enforced social isolation encouraged people to reduce unnecessary activities such as working, studying, shopping, and entertainment. In a bid to accommodate this lifestyle change, online activities have replaced daily routine activities (4).

Furthermore, COVID-19 has raised significant concerns about its impact on health and wellbeing (4). However, the specific health impacts of social isolation policies on the general population have remained unclear. This study aims to examine the impacts of the COVID-19 pandemic on cardiometabolic health, through a retrospective cohort that took physical examinations between 2019 and 2021.

## Materials and Methods

### Study Design

A retrospective cohort study was conducted using historical medical records to compare the COVID-19 pandemic impact on cardiometabolic markers (comparing cardiometabolic markers before the outbreak of COVID-19 with 1 year after the start of the outbreak). This study was approved by the Ethics Committee of the Second Affiliated Hospital of Chongqing Medical University and conducted on the principles of the Helsinki Declaration (No. 2020-252).

### Participants

Adults (without non-communicable diseases) aged 18 years and over underwent medical examinations at the Second Affiliated Hospital of Chongqing Medical University in 2019 and 2020. Medical check-up records from January 2019 to December 2020 were retrieved from the hospital information system (HIS). As the Medical Health Center was almost closed from February to March 2020 due to the COVID-19 outbreak in China, those who had health examinations before April were also excluded from the analysis.

### Data Collection

The anthropometric data (weight, height, and waist and hip circumferences) and blood pressure were measured by trained staff following standard procedures. Bodyweight (kg) and height (cm) were measured in light clothing and without shoes using calibrated digital scales and stadiometers. Body mass index (BMI) was calculated as the weights and heights of participants. Waist and hip circumferences were measured in centimeters with a soft tape scale while participants were standing and wearing no heavy outer garments. Waist circumference (WC) was measured at the level of the umbilicus, and hip circumference was measured at the level of the greater trochanters. Waist-to-hip ratio (WHR) was computed as WC divided by hip circumference. An Omron digital monitor was used for the automated measurement of blood pressure and heart rate.

In this study, blood samples were collected *via* venesection between 7:30 am and 12:00 am after the participant had fasted at least 12 h prior. All blood samples were used to perform biochemical analyses using standard laboratory procedures. These analyses included plasma glucose (mmol/L), glycated hemoglobin (HbA1c, %), high-density lipoprotein cholesterol (HDL-C, mmol/L), low-density lipoprotein cholesterol (LDL-C, mmol/L), total serum cholesterol (TC, mmol/L), triglyceride (TG, mmol/L) levels, plasma uric acid (μmol/L), aspartate aminotransferase (IU/L), and alanine aminotransferase (IU/L).

### Data Analysis

Continuous variables are presented as means ± *SD*s and categorical variables are described with frequency and percentages. Mean paired differences of each participant were compared with zero. The *P*-value was assessed using the Independent sample *t*-test for quantitative variables and the chi-square test for qualitative variables. The paired t-test was used to compare variables between 2019 and 2020 in raw conditions, and the analysis of covariance (ANCOVA) test was used to compare variables between 2019 and 2020 by adjusting covariates. A multiple linear regression model was used to evaluate the association between exposure time of the COVID-19 pandemic and cardiometabolic markers (after adjustment for age, gender, and height). Statistical significance was set at *p* < 0.05. All analyses were performed with the SPSS 26 statistical software package (SPSS Inc, Chicago, IL, USA).

## Results

### Characteristics of the Established Retrospective Cohort

A total of 327,879 records (129,046 in 2019 and 198,833 in 2020) were retrieved, and 31,788 persons with the matched ID number in 2019 and 2020 were selected. After excluding those with age <18 and those with missing or outlier data, 29,773 participants were included in the study. In this cohort, 16,821 (56.12% of the total) participants were men, and the mean age of participants was 42.5 ± 13.44 years and the mean follow-up time was 12.7 ± 2.8 months. Other characteristics and baseline measurements in 2019 are presented in Table 1.

**Table 1:** Characteristics of participants at baseline of 2019.

**Variables**	***N***	**Mean ±SD**	**Male**		**Female**	
			***N***	**Mean ±SD**	***N***	**Mean ±SD**
Age (year)	29,973	42.50 ± 13.44	16,821	42.00 ± 13.11	13,152	43.15 ± 13.81
Height (cm)	29,973	164.58 ± 8.21	16,821	169.59 ± 6.11	13,152	158.16 ± 5.68
Weight (kg)	29,973	63.93 ± 11.89	16,821	70.61 ± 10.25	13,152	55.38 ± 7.61
BMI (kg/m^2^)	29,973	23.52 ± 3.26	16,821	24.53 ± 3.13	13,152	22.15 ± 2.94
Waist (cm)	27,271	80.24 ± 9.95	15,385	85.15 ± 8.30	11,886	73.89 ± 8.17
Hip (cm)	27,265	93.78 ± 6.32	15,381	96.11 ± 5.69	11,884	90.77 ± 5.81
Waist hip ratio	27,265	0.85 ± 0.07	15,381	0.89 ± 0.06	11,884	0.81 ± 0.06
SBP (mmHg)	29,938	120.01 ± 16.68	16,814	123.69 ± 15.55	13,124	115.31 ± 16.88
DBP (mmHg)	29,938	73.05 ± 11.12	16,814	76.06 ± 11.09	13,124	69.21 ± 9.86
FBG (mmo/L)	25,793	5.02 ± 1.07	14,699	5.11 ± 1.22	11,094	4.89 ± 0.81
HbA1c (%)	31,53	5.77 ± 0.89	1,890	5.81 ± 0.96	1,263	5.71 ± 0.77
TC (mmo/L)	27,895	4.96 ± 0.91	15,990	4.94 ± 0.90	11,905	4.98 ± 0.91
TG (mmo/L)	27,896	1.61 ± 1.44	15,990	1.89 ± 1.72	11,906	1.23 ± 0.78
HDL (mmo/L)	22,043	1.37 ± 0.31	12,105	1.28 ± 0.27	9,938	1.49 ± 0.31
LDL (mmo/L)	22,043	2.49 ± 0.67	12,105	2.57 ± 0.67	9,938	2.39 ± 0.66
UA (μmol/L)	28,175	349.62 ± 93.45	16,029	398.03 ± 82.99	12,146	285.73 ± 63.20
AST (IU/L)	26,835	21.77 ± 12.36	15,389	23.49 ± 14.47	11,446	19.46 ± 8.21
ALT (IU/L)	29,251	24.44 ± 25.32	16,602	30.00 ± 30.79	12,649	17.15 ± 12.01

*BMI, body mass index; WHR, waist to hip ratio, SBP, systolic blood pressure; DBP, diastolic blood pressure; TG, triglyceride; TC, total cholesterol; HDL, high-density lipoprotein; LDL, low-density lipoprotein; FBG, fast blood glucose; HbA1c, hemoglobin A1c; UA, uric acid; AST, aspartate aminotransferase; ALT, alanine aminotransferase*.

### COVID-19 Pandemic and Cardiometabolic Markers

The paired differences in cardiometabolic markers in the same person between 2020 and 2019 are presented in Table 2. The raw data showed that weight, BMI, waist circumference, and hip circumference, systolic blood pressure, fasting blood glucose, HbA1c, blood lipids (HDL decreased), uric acid increased (*P* < 0.05) in this cohort due to exposure to COVID-19 pandemic; no significant change was observed for the two liver enzymes (*P* > 0.05). Unlike ALT and AST (*P* > 0.05), the adjusted analysis showed that weight, BMI, waist circumference, hip circumference, WHR, fasting blood glucose, TG, LDL, uric acid, and liver enzymes increased (*P* < 0.05), whereas HDL levels decreased significantly (*P* < 0.05) in this cohort due to exposure to COVID-19 pandemic.

**Table 2:** Paired difference of cardiometabolic markers between 2019 and 2020.

**Variables**	***N***	**Paired difference**	**SD**	*P* value^*^	***P* value^#^**
Weight	29,973	0.40	2.71	<0.001	<0.001
BMI	29,973	0.15	0.99	<0.001	<0.001
Waist	15,112	0.24	5.77	<0.001	<0.001
Hip	15,109	0.27	4.52	<0.001	<0.001
WHR	15,109	<0.0012	0.05	0.671	0.04
SBP	29,921	0.67	12.93	<0.001	0.06
DBP	29,922	−0.15	9.24	0.005	0.01
Glu	24,794	0.02	0.71	<0.001	0.007
HbA1c	1,224	0.04	0.57	0.008	0.08
TC	26,311	0.02	0.68	<0.001	0.73
TG	26,312	0.03	1.26	<0.001	<0.001
HDL	20,295	−0.02	0.22	<0.001	<0.001
LDL	20,295	0.30	0.50	<0.001	<0.001
UA	27,220	10.33	55.07	<0.001	0.002
AST	25,669	0.01	15.10	0.910	<0.001
ALT	28,565	0.17	28.73	0.311	<0.001

* *P-value calculated with paired samples test*.

# *P-value calculated with ANCOVA and the weight, BMI, waist, hip, and WHR adjusted for age and sex and other outcomes adjusted for age, sex, and BMI*.

*BMI, body mass index; WHR, waist to hip ratio, SBP, systolic blood pressure; DBP, diastolic blood pressure; TG, triglyceride; TC, total cholesterol; HDL, high-density lipoprotein; LDL, low-density lipoprotein; FBG, fast blood glucose; HbA1c, hemoglobin A1c; UA, uric acid; AST, aspartate aminotransferase; ALT, alanine aminotransferase*.

**Figure 1:**
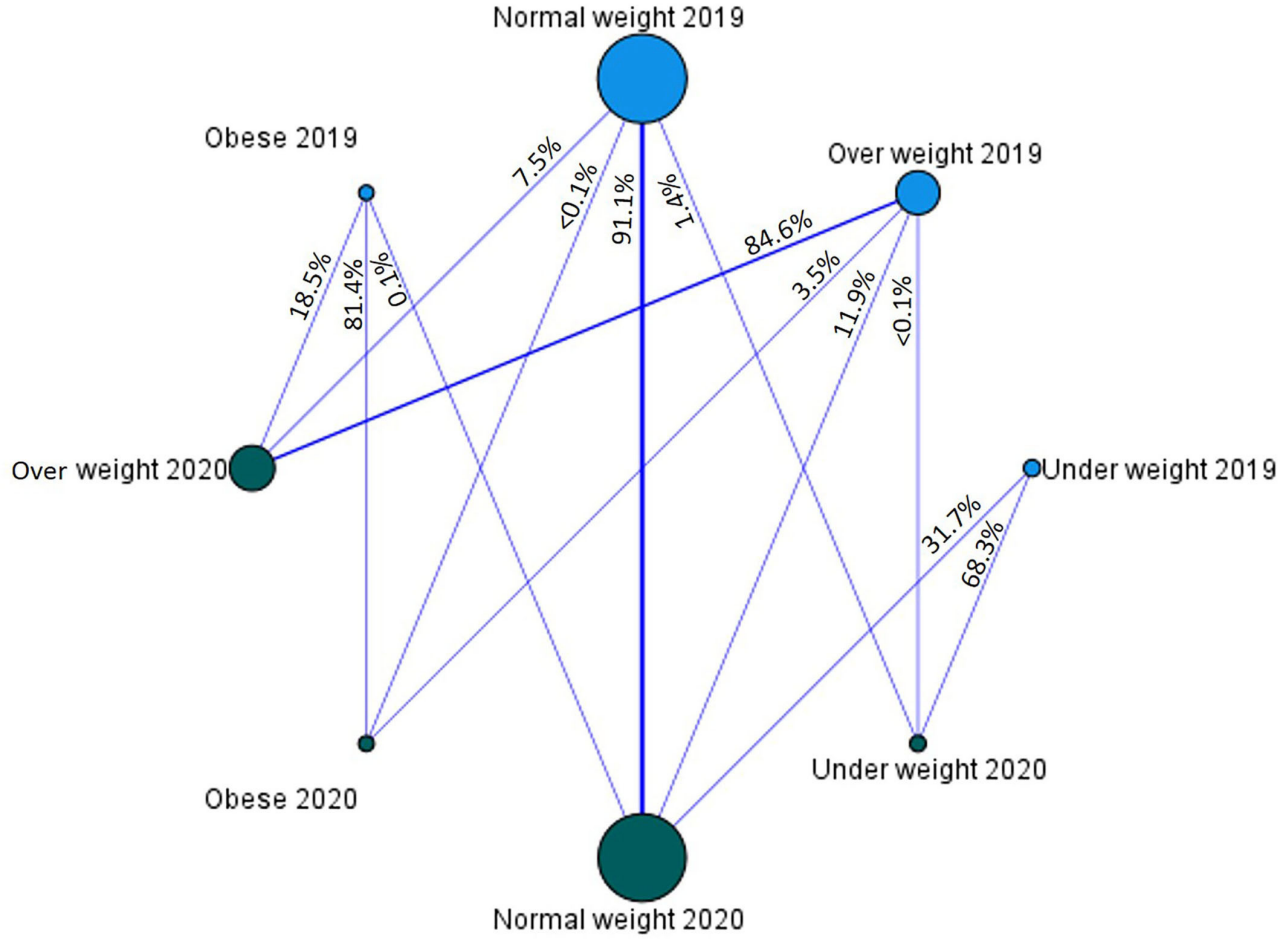
The flow graph of BMI categories between 2019 to 2020 (*P* value < 0.001).

The BMI category flow graph between 2019 and 2020 is provided in Figure 1. It showed that 7.5% and <1% of the normal-weight people in 2019 followed to overweight and obese categories in 2020, respectively. Furthermore, 3.5% of overweight people in 2019 flowed to the obese category in 2020.

### The Duration of COVID-19 Pandemic Exposure and Cardiometabolic Markers

Duration of exposure to COVID-19 was measured in months. Multiple linear regression analysis was performed to test the relationship between exposure duration and cardiometabolic markers. Results showed that exposure was positively associated with weight, BMI, systolic blood pressure, and uric acid (*P* < 0.05), while as negatively related to changes in HDL and AST (*P* < 0.05), with no effects on the waist, DBP, TG, and ALT (*P* > 0.05) (Table 3).

**Table 3:** Multiple linear regression analysis exposure length of COVID-19 and paired changes of cardiometabolic markers.

**Variables**	*B* ^*^	**SD**	***P* value**
Weight	0.125	0.008	<0.001
BMI	0.046	0.003	<0.001
Waist	0.005	0.025	0.854
Hip	0.070	0.019	<0.001
WHR	−0.001	0.001	0.011
SBP	0.349	0.038	<0.001
DBP	−0.047	0.027	0.078
FBG	0.008	0.002	0.001
HbA1c	0.034	0.009	<0.001
TC	0.033	0.002	<0.001
TG	−0.007	0.005	0.113
HDL	−0.007	0.001	<0.001
LDL	0.059	0.002	<0.001
UA	0.681	0.175	<0.001
AST	−0.122	0.048	0.010
ALT	−0.142	0.087	0.104

B^*^: adjusted regression coefficient of exposure length of COVID-19 and cardiometabolic markers. Sex, age, and height were adjusted.

*BMI, body mass index; WHR, waist to hip ratio, SBP, systolic blood pressure; DBP, diastolic blood pressure; TG, triglyceride; TC, total cholesterol; HDL, high-density lipoprotein; LDL, low-density lipoprotein; FBG, fast blood glucose; HbA1c, hemoglobin A1c; UA, uric acid; AST, aspartate aminotransferase; ALT, alanine aminotransferase*.

### Impact of COVID-19 Pandemic Exposure on Cardiometabolic Markers According to Gender

**Figure 2:**
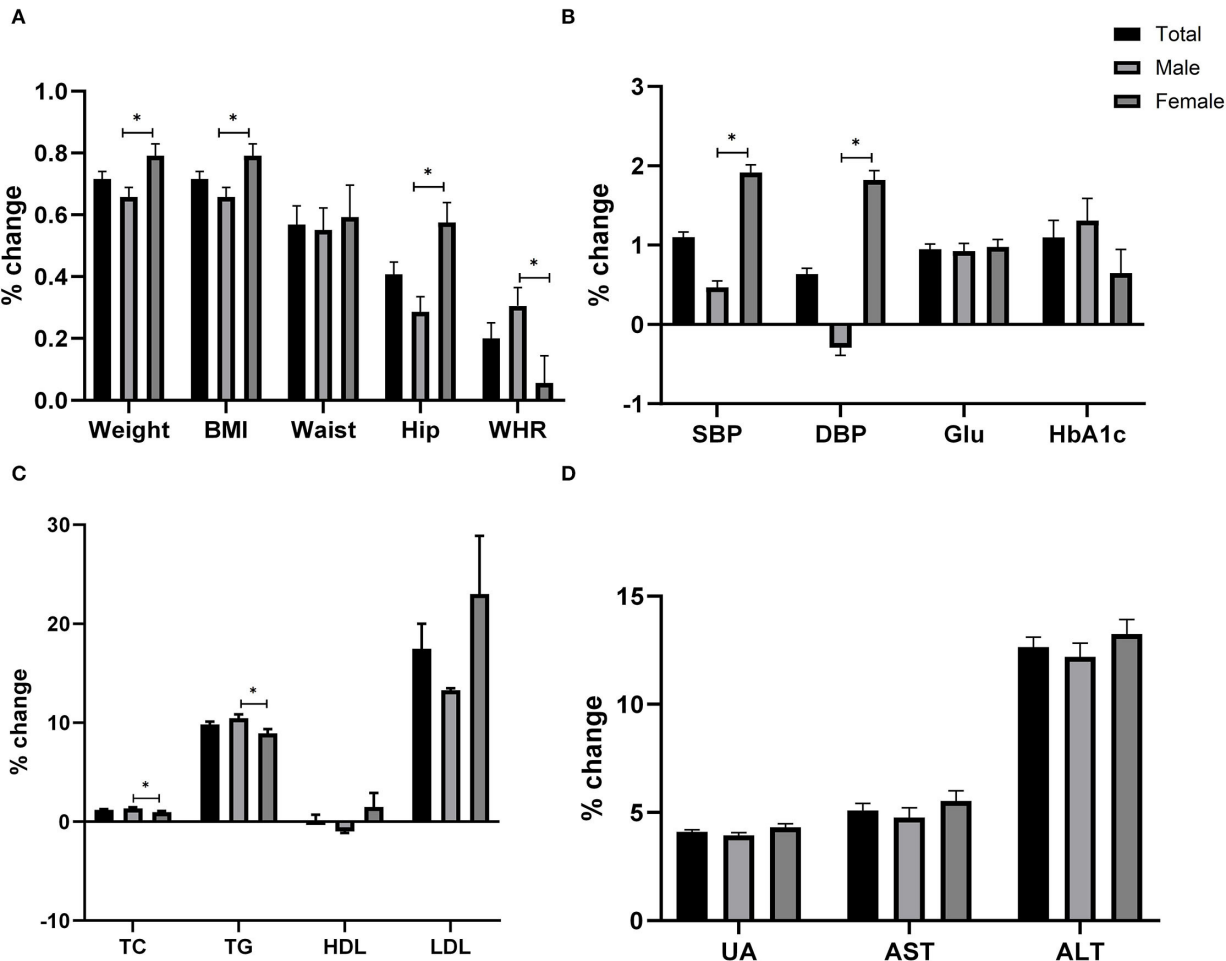
Covid-19 pandemic exposure on cardiometabolic indexes according to sex difference.

Figure 2 provides a percent chance of cardiometabolic markers according to sex difference in 2020 rather than 2019. The weight, BMI, waist, hip, WHR, and other outcomes were adjusted for age and sex. These results showed that weight, BMI, systolic blood pressure (SBP), and diastolic blood pressure (DBP) increased in females more than in males (*P* < 0.05) during pandemic exposure, but WHR, TC, and TG were higher in males compared to female (*P* < 0.05).

### COVID-19 Pandemic Exposure to Cardiometabolic Markers According to BMI Categories

**Figure 3:**
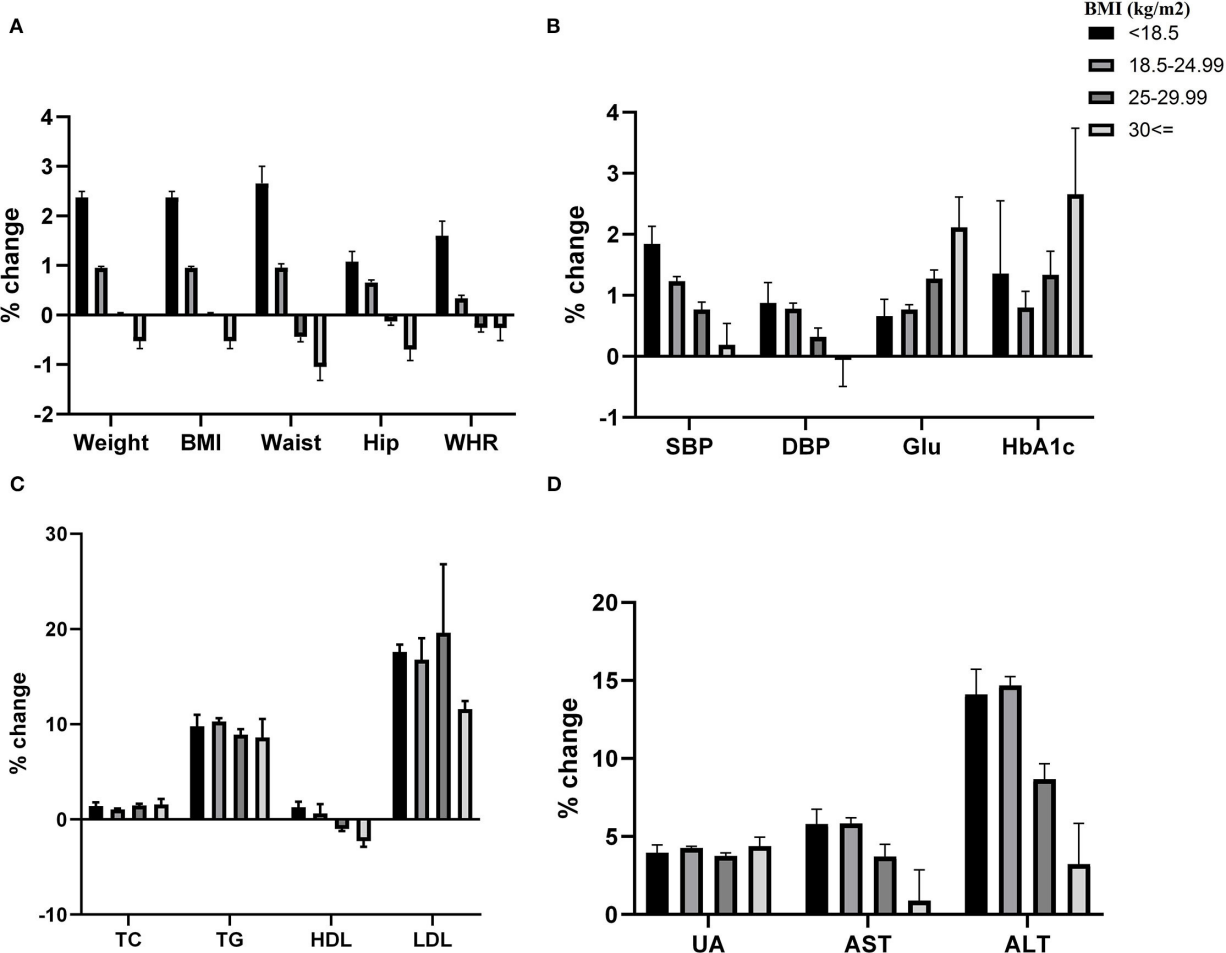
Covid-19 pandemic exposure on cardiometabolic indexes according to BMI categories. Table provided Adjusted *P* value between different levels of BMI to each variable.

Figure 3 provided a percent change in cardiometabolic markers according to BMI categories in 2020 rather than 2019. The weight, BMI, waist, hip, and WHR are adjusted for age and sex, and other outcomes are adjusted for age, sex, and BMI. These results showed that the increase in weight, BMI, waist, hip, WHR, SBP, AST, and ALT are highest in lower BMI categories compared to those with higher BMI (*P* < 0.05). Whereas the rise in fasting blood glucose is more significant in participants with higher BMI compared to those with lower BMI (*P* < 0.05).

### Age Subgroup Analysis of COVID-19 Pandemic Exposure on Cardiometabolic Markers

Results showed that pandemic exposure increased weight, waist circumference, and hip circumference in the youngest age groups (*P* < 0.05). However, SBP, fasting blood sugar, HDL, and uric acid in the oldest age group deteriorated more significantly (*P* < 0.05) (Table 4).

**Table 4:** Paired cardiometabolic markers differences between 2020 and 2019 in different age groups (year in 2020).

**Variables**	**18** **≤ year of age** **<** **40**			**40** **≤ year of age** **<** **60**			**≥60 year of age**		
	***N***	**Mean ±SD**	***P***	***N***	**Mean ±SD**	***P***	***N***	**Mean ±SD**	*P* ^*^
Weight	15,173	0.61 ± 2.94	<0.001	11,124	0.25 ± 2.45	<0.001	3,676	0.01 ± 2.42	0.838
BMI	15,173	0.22 ± 1.06	<0.001	11,124	0.10 ± 0.91	<0.001	3,676	0.004 ± 0.94	0.787
Waist	7,698	0.37 ± 5.94	<0.001	5,743	0.22 ± 5.49	0.002	1,671	−0.28 ± 5.89	0.053
Hip	7,698	0.33 ± 4.71	<0.001	5,740	0.32 4.24	<0.001	1,671	−0.13 ± 4.58	0.242
WHR	7,698	0.001 ± 0.05	0.079	5,740	<0.0014 ± 0.05	0.508	1,671	−0.001 ± 0.06	0.246
SBP	15,156	0.02 ± 11.73	0.797	11,103	1.05 ± 13.31	<0.001	3,662	2.20 ± 15.97	<0.001
DBP	15,157	−0.22 ± 8.70	0.002	11,103	0.10 ± 9.49	0.273	3,662	−0.61 ± 10.51	<0.001
FBG	12,352	0.01 ± 0.52	0.017	9,451	0.03 ± 0.77	0.001	2,991	0.06 ± 1.11	0.003
HbA1c	173	−0.09 ± 0.76	0.117	782	0.08 ± 0.52	<0.001	269	0.01 ± 0.54	0.745
TC	12,855	0.00 ± 0.16	0.474	10,056	0.05 ± 0.71	<0.001	3,400	0.01 ± 0.82	0.316
TG	12,855	0.03 ± 1.15	0.001	10,057	0.03 ± 1.45	0.022	3,400	0.03 ± 1.03	0.057
HDL	7,9s46	−0.01 ± 0.22	<0.001	9,080	−0.02 ± 0.22	<0.001	3,269	−0.05 ± 0.23	<0.001
LDL	7,946	0.31 ± 0.43	<0.001	9,080	0.31 ± 0.51	<0.001	3,269	0.26 ± 0.62	<0.001
UA	13,496	9.96 ± 55.45	<0.001	10,378	9.28 ± 53.4	<0.001	3,346	15.11 ± 58.32	<0.001
AST	13,142	0.04 ± 15.35	0.777	9,544	−0.05 ± 11.46	0.690	2,983	0.07 ± 22.47	0.858
ALT	14,624	0.27 ± 28.78	0.252	10,580	−0.05 ± 31.57	0.861	3,361	0.45 ± 16.58	0.117

* *Compared with zero*.

*BMI, body mass index; WHR, waist to hip ratio, SBP, systolic blood pressure; DBP, diastolic blood pressure; TG, triglyceride; TC, total cholesterol; HDL, high-density lipoprotein; LDL, low-density lipoprotein; FBG, fast blood glucose; HbA1c, hemoglobin A1c; UA, uric acid; AST, aspartate aminotransferase; ALT, alanine aminotransferase*.

## Discussion

To control the spread of COVID-19, governments pursued lockdown measures to curtail social mobility. Its true cost on cardiovascular and metabolic health remains unclear despite the plethora of literature examining the health impact of the pandemic (5).

In the present study, we established a retrospective cohort with 29,773 participants and compared the cardiometabolic markers before the outbreak of COVID-19 with 1 year after the start of the outbreak. Results showed that there were significant weight increases, waist circumference, hip circumference, blood pressure, fasting blood glucose, lipids, and uric acid in this cohort during the first year of COVID-19 pandemic exposure, which suggested that the pandemic may have worsened the risk of developing cardiovascular and metabolic diseases among the general population.

The adverse effects of social isolation and change in lifestyle brought about by pandemic exposure may have contributed to worsening cardiometabolic markers. In the first outbreak, China experienced a 3-month-long national lockdown to break the chain of transmission in the community (6). After lifting the national lockdown, all kinds of social activities were discouraged, and stay-at-home lifestyles were promoted. Studies examining behaviors in the Chinese population have shown that more time was spent on electronic screens in preschool children (7), in youths (8), increased snack intake, reduced physical activity, and sleep duration in adults (9–11). Physical activity is an essential factor in physical and mental health (12) and is strongly recommended for health and wellbeing (13). Therefore, reduced physical activity during the pandemic can explain the worsening metabolic health indicators examined in this study. Furthermore, the COVID-19 pandemic also reduced the availability of healthy and fresh foods (14), contributing to the worsening of cardiometabolic markers in our study. Other studies also indicated that during the pandemic, people suffered economic pressure and mental health problems (15), which may reversely reduce the quality of food and lead to the deterioration of cardiometabolic parameters.

The findings of our study are consistent with the other two published longitudinal studies (16, 17). In our study, the average follow-up interval was over 12 months. Additionally, we examined the association between COVID-19 pandemic exposure time and changes in cardiometabolic markers, which confirms the results of other studies with more reliability.

In this present study, an age subgroup analysis explored the impact of COVID-19 on different age groups. Results showed that weight gain-related parameters were worse in the younger group than in the older groups. Metabolic indexes such as SBP, fasting blood glucose, and uric acid was worse in the older groups. This may be due to older adults being predisposed to cardiovascular and metabolic diseases.

The main advantage of this study is the large sample size and longitudinal cohort design, which can provide robustness and reliability. The limitations of this study include this study population originating from one center, which may introduce selective bias. Secondly, due to the lack of social and economic information, such as education, occupation, and income, it is impossible to estimate the effects of these confounding factors. Lastly, behavior and emotional data were unavailable in this study, such as diet and exercise. Therefore, we cannot provide a direct explanation for our findings. One of the other limitations of this study is maturation bias. Maturation bias occurs when natural changes over time, like increasing age, may influence the study outcomes.

In conclusion, this study showed that the COVID-19 pandemic and its control measures significantly negatively impacted cardiometabolic profiles, especially in older adults. The result of this study may help promote a healthier lifestyle to cope with the unwanted effects of COVID-19 pandemic measures.

## Data Availability Statement

The raw data supporting the conclusions of this article will be made available by the authors, without undue reservation.

## Ethics Statement

This study was approved by the Ethics Committee of the Second Affiliated Hospital of Chongqing Medical University and conducted in accordance with the Principles of the Helsinki Declaration (No. 2020-252). Written informed consent was not provided because patient consent was waived due to the fact that this was a retrospective observational study, and anonymized databases provided by the health authorities were used.

## Author Contributions

XQ and ZH: conceptualization. ZH, YZ, and XX: methodology, formal analysis, writing-original draft, writing-review, and editing. YM and JR: software and data curation. SS: visualization. All authors have read and agreed to the published version of the manuscript.

## Funding

This work was supported by the Intelligent Medicine Project of Chongqing Medical University (ZHYX202024).

## Conflict of Interest

The authors declare that the research was conducted in the absence of any commercial or financial relationships that could be construed as a potential conflict of interest.

## Publisher's Note

All claims expressed in this article are solely those of the authors and do not necessarily represent those of their affiliated organizations, or those of the publisher, the editors and the reviewers. Any product that may be evaluated in this article, or claim that may be made by its manufacturer, is not guaranteed or endorsed by the publisher.
